# Head Impact Exposure in Youth Football: Elementary School Ages 9–12 Years and the Effect of Practice Structure

**DOI:** 10.1007/s10439-013-0867-6

**Published:** 2013-07-24

**Authors:** Bryan R. Cobb, Jillian E. Urban, Elizabeth M. Davenport, Steven Rowson, Stefan M. Duma, Joseph A. Maldjian, Christopher T. Whitlow, Alexander K. Powers, Joel D. Stitzel

**Affiliations:** 1 School of Biomedical Engineering & Sciences, Virginia Tech-Wake Forest University, 440 ICTAS Building, Stanger St., Blacksburg, VA 24061 USA; 2 School of Biomedical Engineering & Sciences, Virginia Tech-Wake Forest University, Medical Center Blvd., Winston-Salem, NC 27157 USA; 3 Wake Forest School of Medicine, Medical Center Blvd., Winston-Salem, NC 27157 USA; 4 Department of Radiology (Neuroradiology), Wake Forest School of Medicine, Winston-Salem, NC 27157 USA; 5 Translational Science Institute, Wake Forest School of Medicine, Winston-Salem, NC 27157 USA; 6 Department of Neurosurgery, Wake Forest School of Medicine, Winston-Salem, NC 27157 USA; 7 Childress Institute for Pediatric Trauma, Wake Forest School of Medicine, Winston-Salem, NC 27157 USA

**Keywords:** Concussion, Brain injury, Biomechanics, Helmet, Linear, Rotational, Acceleration, Pediatrics, Children, Sports

## Abstract

Head impact exposure in youth football has not been well-documented, despite children under the age of 14 accounting for 70% of all football players in the United States. The objective of this study was to quantify the head impact exposure of youth football players, age 9–12, for all practices and games over the course of single season. A total of 50 players (age = 11.0 ± 1.1 years) on three teams were equipped with helmet mounted accelerometer arrays, which monitored each impact players sustained during practices and games. During the season, 11,978 impacts were recorded for this age group. Players averaged 240 ± 147 impacts for the season with linear and rotational 95th percentile magnitudes of 43 ± 7 g and 2034 ± 361 rad/s^2^. Overall, practice and game sessions involved similar impact frequencies and magnitudes. One of the three teams however, had substantially fewer impacts per practice and lower 95th percentile magnitudes in practices due to a concerted effort to limit contact in practices. The same team also participated in fewer practices, further reducing the number of impacts each player experienced in practice. Head impact exposures in games showed no statistical difference. While the acceleration magnitudes among 9–12 year old players tended to be lower than those reported for older players, some recorded high magnitude impacts were similar to those seen at the high school and college level. Head impact exposure in youth football may be appreciably reduced by limiting contact in practices. Further research is required to assess whether such a reduction in head impact exposure will result in a reduction in concussion incidence.

## Introduction

In recent years, football has come under increased scrutiny because of the concern for player safety and the risk of injury, especially related to concussion. Researchers estimate that between 1.6 and 3.8 million cases of sports related concussion occur each year in the United States, with football having the highest rate of injury among team sports.^14^,^19^ While the long term effects of sports concussions are still under investigation, links may exist between the accumulation of head impacts over a playing career and increased risk of neurodegenerative diseases later in life, among other health concerns.^30^ The majority of the biomechanics research investigating concussions in football has been focused on high school, college, and professional players, despite that more than two-thirds of football players are under the age of 14.^11^

In order to understand the biomechanics associated with concussion, numerous studies have been conducted over the last decade to investigate player exposure and tolerance to head impacts in football.^3^,^4^,^7^,^9^,^10^,^13^,^16^,^17^,^22^,^23^,^25^–^27^,^31^–^34^ Many of these studies have utilized commercially available helmet-mounted accelerometer arrays (Head Impact Telemetry (HIT) System, Simbex, Lebanon, NH) to measure head kinematics resulting from head impact in real-time during live play. The accelerometer arrays collect data from each head impact a player experiences while instrumented, allowing researchers to get a more complete view of the biomechanical response of a player’s head to impacts across a wide range of magnitudes. Since 2003, more than 1.5 million impacts have been recorded using the HIT system, primarily at the high school and college level.^7^–^10^,^12^ From these data, strategies to reduce head impact exposure through rule changes and methods to evaluate protective equipment have been developed.^8^–^10^,^25^ Unfortunately, little research has focused on youth football, where the head impact exposure is still not well understood.^11^ A single study has investigated head impact exposure at the youth level. That study found that 7 and 8 year old players sustained an average of 107 impacts over the course of a season, with the majority of high magnitude impacts occurring in practice.^11^ This work was one factor contributing to youth football organizations updating contact restrictions during practice.^28^

An estimated 5 million athletes participate in organized football in the United States annually. Children, age 6–13 years, account for around 3.5 million of these participants, compared to just 2000 in the National Football League (NFL), 100,000 in college, and 1.3 million in high school.^11^,^18^,^24^ Despite making up 70% of the football playing population, just one study has investigated head impact exposure experienced by youth football players under 14 years old. The objective of this study was to quantify the head impact exposure of youth football players, aged 9–12 years, for all practices and games over the course of single season. These data, along with future research, may be used in the development of scientifically based strategies for head injury mitigation.

## Materials and Methods

On-field head impact data were collected from 50 players, age 9–12 years, on three youth tackle football teams instrumented with the HIT system for a single fall football season. The three teams consisted of a juniors team (team A, 9–11 years old), a pee wee team (team B, 10–12 years old), and a junior pee wee team (team C, 9–11 years old). Further description of the three teams is provided in Table 1. Players were monitored during each of the teams’ games and contact practices. Approval for this study was given by the Virginia Tech and Wake Forest University Institutional Review Boards (IRBs). Each player provided assent and their parent/guardian gave written consent for participation in the study.

**Table 1:** Description of subject groups investigated in this study

Team	Player mass (kg)	Player age (years)	Number of players	Number of impacts
A	37.6 ± 5.7	9.8 ± 0.8	14	2206
B	50.1 ± 3.9	12.2 ± 0.5	17	5005
C	43.9 ± 5.9	10.9 ± 0.6	19	4767
Combined	44.2 ± 7.2	11.0 ± 1.1	50	11,978

The HIT system consists of an array of six non-orthogonally mounted single-axis accelerometers oriented normal to the surface of the head. The arrays, designed to fit in medium or large Riddell Revolution helmets, were installed between the existing padding inside the helmets. Each accelerometer is mounted on an elastic base so that they remain in contact with the head throughout the duration of head impact, allowing for the measurement of head acceleration rather than that of the helmet.^20^ Any time an instrumented player experienced a head impact that resulted in a single accelerometer measuring 14.4 g during games and practices, data acquisition was triggered to record 40 ms of data at 1000 Hz, including 8 ms of pre-trigger data. Data from the helmet-mounted accelerometers were then transmitted wirelessly to a computer on the sideline, where the data were stored and processed to compute resultant linear head acceleration and peak rotational head acceleration using previously described methods.^6^,^27^ In addition, impact location was generalized into 1 of 4 impact locations (front, side, top, or back) based on the acceleration vectors from the linear accelerometers.^15^ Impacts were verified using video from practice and game sessions to ensure they occurred while players were wearing the helmets. The HIT system has previously been found to reliably determine linear acceleration, peak rotational acceleration, and impact location.^1^

Empirical cumulative distribution functions (CDF) for both linear and rotational head acceleration were determined. Head impact exposure was quantified in terms of impact frequency and 50th and 95th percentile head accelerations. Acceleration duration was measured from the local minimum before peak linear acceleration and the local minimum after the peak, while time to peak linear acceleration was measured from the local minimum before peak linear acceleration to the peak. The data were sorted by generalized impact location and session type (practice or game). A Kruskal–Wallis one-way analysis of variance was conducted to evaluate for between-group differences in head impact exposure associated with the three teams and two session types. A threshold of *p* < 0.05 was used to determine statistical significance. In the event that more than two groups were compared, *p* values were calculated for all pairs and the most conservative *p* value was reported. All data analysis was conducted on an individual player basis and then averaged to represent the exposure level of a typical 9–12 year old football player. Head impact exposure levels were then compared with those of other levels of play that have been previously described in the literature.

## Results

**Figure 1:**
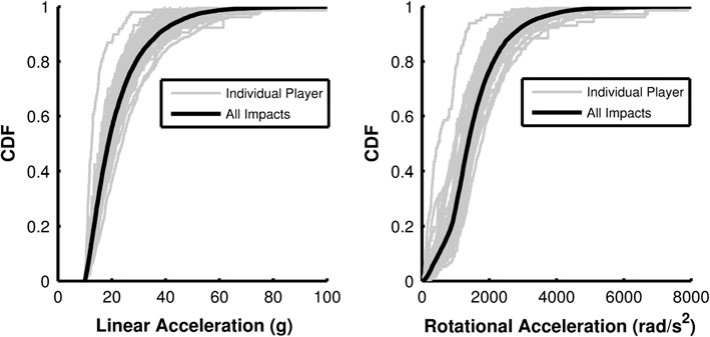
Cumulative distribution plots of linear acceleration (left) and rotational acceleration (right) magnitudes for impacts collected during the season

**Figure 2:**
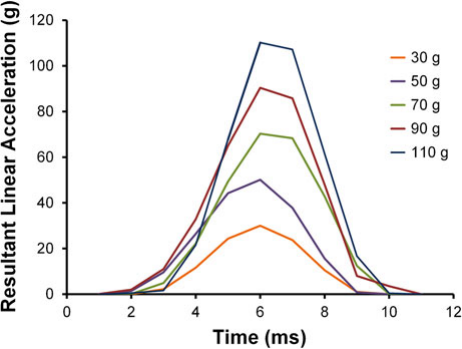
Resultant linear acceleration vs. time for several impacts of various magnitudes recorded from 9 to 12 year old football players

A total of 11,978 impacts were measured, ranging from linear accelerations of 10–126 g and rotational accelerations of 4–5838 rad/s^2^. The distribution of linear acceleration had a median value of 19 g and a 95th percentile value of 46 g. The distribution of rotational acceleration had a median value of 890 rad/s^2^ and a 95th percentile value of 2081 rad/s^2^. CDFs of linear and rotational acceleration magnitudes for the season were determined (Fig. 1). The acceleration distributions are right-skewed and heavily weighted toward lower magnitude impacts. The impact durations measured were 8.82 ± 2.97 ms (average ± standard deviation) with a time to peak linear acceleration of 4.67 ± 1.73 ms. Resultant linear acceleration is plotted vs. time for several impacts recorded in this study as, examples of a typical acceleration pulse (Fig. 2).

On average, instrumented players sustained 240 ± 147 impacts during the season, with values ranging from 26 to 585 impacts. The average instrumented player sustained 10.6 ± 5.2 impacts per session while participating in 21.8 ± 5.7 sessions. The median impact sustained by instrumented players resulted in accelerations of 18 ± 2 g and 856 ± 135 rad/s^2^. The 95th percentile impact sustained by instrumented players resulted in accelerations of 43 ± 7 g and 2034 ± 361 rad/s^2^. Head impact exposure was quantified on an individual player basis by session type (Table 2). A total of 961 impacts (8.0%) greater than 40 g, 160 impacts (1.3%) greater than 60 g, and 36 impacts (0.3%) greater than 80 g were recorded throughout the season. The average player sustained 19.2 ± 20.1 impacts greater than 40 g, 3.2 ± 4.4 impacts greater than 60 g, and 0.7 ± 1.2 impacts greater than 80 g.

**Table 2:** Expanded head impact exposure data for each player, split up by session type for each team: (a) team A, (b) team B, and (c) team C

Player ID	Practice							Games							Season						
	Sessions	Number of Impacts		Linear Acceleration (g)		Rotational Acceleration (rad/s^2^)		Sessions	Number of Impacts		Linear Acceleration (g)		Rotational Acceleration (rad/s^2^)		Sessions	Number of Impacts		Linear Acceleration (g)		Rotational Acceleration (rad/s^2^)	
		Total	Per Session	50%	95%	50%	95%		Total	Per Session	50%	95%	50%	95%		Total	Per Session	50%	95%	50%	95%
(a)																					
A1	8	53	6.6	14	26	319	982	6	46	7.7	11	17	269	1004	14	99	7.1	13	25	305	1051
A2	3	19	6.3	15	34	667	1247	7	73	10.4	20	53	973	2401	10	92	9.2	17	54	890	2348
A3	8	52	6.5	21	41	996	2019	8	176	22	20	42	940	2139	16	228	14.3	20	42	943	2130
A4	10	87	8.7	16	42	448	1915	7	286	40.9	19	46	702	2186	17	373	21.9	18	45	633	2156
A5	9	36	4	16	33	647	1321	6	25	4.2	15	44	603	2650	15	61	4.1	16	35	637	1826
A6	10	53	5.3	19	30	770	1530	8	54	6.8	18	33	903	1826	18	107	5.9	19	33	854	1570
A7	8	44	5.5	16	27	642	1489	7	45	6.4	18	35	838	1474	15	89	5.9	17	30	785	1494
A8	9	39	4.3	17	38	698	1891	7	109	15.6	18	45	791	1883	16	148	9.3	18	44	777	1892
A9	7	16	2.3	16	29	669	1392	8	56	7	21	58	1114	3310	15	72	4.8	20	53	1002	3115
A10	7	78	11.1	19	32	669	1509	7	160	22.9	17	32	633	1778	14	238	17	17	32	664	1738
A11	9	80	8.9	16	30	714	1227	6	160	26.7	16	43	811	2252	15	240	16	16	36	780	1796
A12	9	56	6.2	17	36	752	1461	8	96	12	19	39	813	2068	17	152	8.9	18	38	774	1953
A13	5	26	5.2	16	33	670	1541	8	61	7.6	18	48	576	2151	13	87	6.7	17	47	613	1724
A14	6	35	5.8	17	32	728	1455	8	185	23.1	18	36	808	1696	14	220	15.7	18	35	788	1696
Ave.	7.7	48	6.2	17	33	671	1499	7.2	109	15.2	18	41	770	2059	14.9	158	10.5	17	39	746	1892
SD	1.9	21	2.1	2	5	147	274	0.8	71	10.1	2	10	199	524	1.9	87	5.3	2	8	166	456
(b)																					
B1	18	104	5.8	19	34	913	1944	6	9	1.5	23	46	983	2183	24	113	4.7	19	39	920	2077
B2	15	129	8.6	17	48	866	1769	7	21	3	22	35	879	1929	22	150	6.8	18	47	874	1796
B3	15	114	7.6	20	41	1018	2020	7	21	3	17	36	865	2169	22	135	6.1	19	41	1011	2072
B4	21	338	16.1	21	58	1004	2732	9	199	22.1	25	64	1121	2907	30	537	17.9	22	59	1061	2801
B5	13	146	11.2	21	42	1045	2164	5	25	5	18	41	705	1627	18	171	9.5	19	42	994	2083
B6	17	248	14.6	18	45	980	2152	7	74	10.6	21	44	1051	2097	24	322	13.4	19	46	988	2154
B7	8	107	13.4	19	42	864	1660	5	45	9	21	48	923	2342	13	152	11.7	19	48	895	1974
B8	18	314	17.4	22	44	974	2159	9	84	9.3	20	45	856	2235	27	398	14.7	21	44	956	2177
B9	15	87	5.8	16	32	788	1606	9	50	5.6	18	36	938	1675	24	137	5.7	17	33	835	1629
B10	15	197	13.1	19	45	861	2059	8	218	27.3	21	50	977	2605	23	415	18	19	48	924	2437
B11	14	128	9.1	19	37	969	1693	8	37	4.6	18	35	917	1795	22	165	7.5	18	37	964	1718
B12	18	423	23.5	21	49	900	2001	8	87	10.9	21	48	975	1989	26	510	19.6	21	49	904	2005
B13	21	484	23	24	49	1170	2474	8	101	12.6	21	51	1044	2136	29	585	20.2	24	49	1137	2437
B14	18	341	18.9	17	42	734	1901	8	98	12.3	17	48	753	2113	26	439	16.9	17	42	743	1918
B15	16	116	7.3	18	38	859	2151	7	27	3.9	18	36	888	1958	23	143	6.2	18	38	881	2128
B16	18	192	10.7	16	35	834	1808	7	40	5.7	18	46	923	2411	25	232	9.3	16	37	837	1881
B17	19	246	12.9	19	48	904	2177	7	155	22.1	20	52	935	2459	26	401	15.4	19	50	915	2379
Ave.	16.4	218	12.9	19	43	923	2028	7.4	76	9.9	20	45	925	2155	23.8	294	12	19	44	932	2098
SD	3	119	5.4	2	6	102	282	1.2	61	7.3	2	7	99	319	3.9	159	5.2	2	6	90	284
(c)																					
C1	18	258	14.3	19	43	931	1873	6	67	11.2	20	45	989	1954	24	325	13.5	19	44	940	1951
C2	22	377	17.1	20	47	1012	2511	8	191	23.9	23	49	1152	2411	30	568	18.9	21	47	1050	2463
C3	18	152	8.4	16	37	825	1766	9	36	4	16	37	837	1537	27	188	7	16	37	835	1690
C4	17	125	7.4	18	41	858	1866	7	85	12.1	18	44	821	2449	24	210	8.8	18	43	850	2179
C5	18	143	7.9	17	38	842	2137	8	56	7	16	31	748	1687	26	199	7.7	16	36	818	1911
C6	21	286	13.6	17	46	852	2164	9	244	27.1	20	47	945	2128	30	530	17.7	19	47	898	2153
C7	17	154	9.1	18	39	874	1765	7	30	4.3	19	32	852	1444	24	184	7.7	18	38	874	1745
C8	18	125	6.9	16	37	757	1519	5	19	3.8	17	50	731	2654	23	144	6.3	16	38	755	1844
C9	18	171	9.5	18	47	925	2321	8	33	4.1	17	27	882	1624	26	204	7.8	18	46	921	2276
C10	21	283	13.5	17	40	801	1587	8	114	14.3	19	40	851	1895	29	397	13.7	18	40	821	1652
C11	17	187	11	17	37	823	1778	8	122	15.3	19	34	888	1772	25	309	12.4	18	36	845	1783
C12	20	148	7.4	17	51	745	2196	9	55	6.1	17	43	783	2224	29	203	7	17	49	759	2206
C13	16	155	9.7	18	47	876	2190	7	84	12	19	51	1001	1926	23	239	10.4	19	48	957	2073
C14	19	210	11.1	18	40	894	2190	8	30	3.8	18	47	976	2662	27	240	8.9	18	40	899	2254
C15	21	122	5.8	19	54	929	2705	9	26	2.9	18	50	824	2387	30	148	4.9	19	54	885	2715
C16	18	268	14.9	20	42	985	2100	9	177	19.7	19	47	921	2307	27	445	16.5	20	44	946	2140
C17	15	66	4.4	21	48	1013	2180	8	46	5.8	20	51	913	2344	23	112	4.9	21	50	963	2367
C18	13	81	6.2	16	36	765	1464	5	15	3	20	37	967	1724	18	96	5.3	17	37	780	1601
C19	7	17	2.4	15	47	681	2456	5	9	1.8	15	57	777	3259	12	26	2.2	16	56	742	2565
Ave.	17.6	175	9.5	18	43	863	2040	7.5	76	9.6	18	43	887	2126	25.1	251	9.5	18	44	870	2083
SD	3.3	85	3.8	2	5	89	334	1.4	64	7.3	2	8	101	451	4.3	141	4.6	1	6	80	311

In games, the average player had a median linear acceleration value of 19 ± 2 g and a 95th percentile value of 43 ± 8 g. The average player had a median linear acceleration value of 18 ± 2 g and 95th percentile value of 40 ± 7 g in practices. Both the difference in median (*p* = 0.0289) and 95th percentile (*p* = 0.0463) linear acceleration magnitudes between games and practices were significant. For rotational acceleration, the average player had a median value of 867 ± 149 rad/s^2^ and a 95th percentile value of 2117 ± 436 rad/s^2^ for games. In practices, the average player had a median rotational acceleration value of 829 ± 152 rad/s^2^ and a 95th percentile value of 1884 ± 385 rad/s^2^. As with linear acceleration, the difference between game and practice 95th percentile rotational acceleration (*p* = 0.0099) was significant. The average player sustained 154 ± 113 impacts in 14.4 ± 5.2 contact practices and 85 ± 68 impacts in 7.4 ± 1.2 games. On a per session basis, players experienced 9.7 ± 4.9 impacts per practice and 11.3 ± 8.7 impacts per game. While players experienced significantly more impacts in practices than games (*p* = 0.0011) throughout the season, the difference in the number of impacts per session for practices and games (*p* = 0.9423) was not significant.

**Figure 3:**
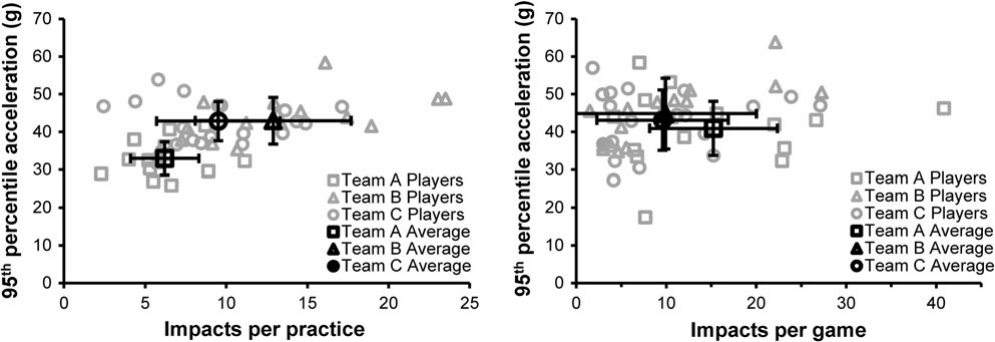
Player 95th percentile acceleration magnitude vs. number of impacts per session for practices (left) and games (right). Individual players are shown in gray while team averages are displayed in black with error bars showing standard deviation

Substantial differences existed among the three teams in this study for both impact frequency and acceleration magnitude (Table 3). Players on team A accumulated fewer impacts in practices during the season (*p* < 0.0001) than those on teams B and C, as well as fewer impacts on a per practice basis (*p* < 0.0097). Furthermore, team A players sustained appreciably lower magnitude accelerations than their team B and C counterparts (Fig. 3). For linear acceleration magnitude, the 95th (*p* < 0.0001) percentile differences between team A and the other two was significant for practices. Likewise, the difference in rotational acceleration magnitudes between team A and teams B and C was significant for the median (*p* < 0.0001) and 95th percentile (*p* < 0.002) values for practices. In games, impact frequency and acceleration magnitudes were not significantly different among the teams. Team A players sustained significantly fewer impacts throughout the season compared to team B players (*p* = 0.0045) due to practice differences. While team A players also sustained fewer impacts during the season than team C players, the difference was not significant (*p* = 0.0742).

**Table 3:** Summary comparison of three teams of 9–12 year old players

Team	Practices						Games						Season					
	Impacts		Linear acceleration (g)		Rotational acceleration (rad/s^2^)		Impacts		Linear acceleration (g)		Rotational acceleration (rad/s^2^)		Impacts		Linear acceleration (g)		Rotational acceleration (rad/s^2^)	
	Total	Per session	Median (50%)	95%	Median (50%)	95%	Total	Per session	Median (50%)	95%	Median (50%)	95%	Total	Per session	Median (50%)	95%	Median (50%)	95%
A	48	6.2	17	33	671	1499	109	15.2	18	41	770	2059	158	10.5	17	39	746	1892
B	218	12.9	19	43	923	2028	76	9.9	20	45	925	2155	294	12.0	19	44	932	2098
C	175	9.5	18	43	863	2040	76	9.6	18	43	887	2126	251	9.5	18	44	870	2083

Impacts to the front of the helmet were the most common, representing 41% of all impacts, followed by those to the back at 25% and side at 23% (Table 4). The least frequently impacted location was the top of the helmet, representing 11% of all impacts. Impacts to the top of the helmet resulted in the highest magnitude linear accelerations with a median value of 21 g and a 95th percentile value of 46 g. For rotational acceleration, impacts to the front had the highest values while those to the top had the lowest.

**Table 4:** Head impact frequency and magnitude by location for 9–12 year old players

Location	Percentage of impacts (%)	Linear acceleration (g)		Rotational acceleration (rad/s^2^)	
		50th	95th	50th	95th
Front	52	19	41	951	2049
Side	19	16	34	810	1715
Rear	18	18	41	790	2030
Top	10	21	46	388	1040

Among the three teams participating in this study, four instrumented players sustained concussions diagnosed by physicians: two on the pee wee team (B4 and B6) and one on each of the other two teams (A8 and C18). The impact associated with player A8’s concussion was to the front of the helmet and had a linear acceleration of 58 ± 9 g and rotational acceleration of 4548 ± 1400 rad/s^2^. For player B4, the concussion was associated with an impact to the back of the helmet with linear and rotational acceleration magnitudes of 64 ± 10 g and 2830 ± 900 rad/s^2^. No impacts were recorded for B6 on the day of his concussion due to a battery failure in the sensor array. Player C18’s concussion was linked to an impact to the side of the helmet with linear and rotational acceleration magnitudes of 26 ± 4 g and 1552 ± 500 rad/s^2^.

## Discussion

Previous studies have investigated the frequency and magnitude of head impacts in other tackle football populations, including youth (7–8 years), high school (14–18 years), and college (18–23 years) in the last decade (Table 5).^5^,^11^,^25^,^27^ Data from these studies show a trend of increasing acceleration magnitude and impact frequency with increasing level of play. Not surprisingly, the 9–12 year old players in this study were found to experience linear acceleration magnitudes between those found in 7–8 year old players and high school players. For rotational acceleration, the 95th percentile magnitude found in this study was less than that found previously in younger players.^11^ Rotational acceleration tends to correlate well with linear acceleration, though impact location can heavily influence the relationship.^27^ Players in this study experienced more impacts to the front of their helmets and fewer to the side than the 7–8 year old players studied by Daniel *et al*.^11^ In that study, impacts to the front of player’s helmets were associated with lower rotational acceleration magnitudes, while those to the side were associated with higher magnitudes.

**Table 5:** Comparison of head impact exposure across various levels of play^3^,^5^,^11^,^25^,^27^

Level of play	Number of impacts per season	Linear acceleration (g)		Rotational acceleration (rad/s^2^)	
		Median (50%)	95%	Median (50%)	95%
Youth (7–8 years)	107	15	40	672	2347
Youth (9–12 years)	240	18	43	856	2034
High school (14–18 years)	565	21	56	903	2527
College (19–23 years)	1000	18	63	981	2975

As with magnitude, the impact frequency reported in this study fell between those of 7–8 year old and high school athletes. In this study, the average player experienced 240 impacts throughout the season compared to 107 impacts per season for 7–8 year old players and 565 for high school players.^3^,^5^,^11^ This trend can be partially attributed to the number of sessions (practices and games) increasing as the level of play increases. The 7–8 year old team studied by Daniel *et al*.^11^ experienced impacts in 9.4 practices and 4.7 games for a total of 14.1 sessions. Players in this study participated in an average of 14.4 contact practices and 7.4 games, for a total of 21.8 sessions. Compared to the high school team studied by Broglio *et al*.,^3^ the teams in this study participated in fewer practices and games in addition to experiencing fewer impacts per session. High school players experienced on average 15.9 impacts per session whereas the 9–12 year old players in this study experienced 10.6 impacts per session. The age related differences reported among these three age groups are most likely due to increased size, athleticism, and aggression in older players.

Players experienced slightly greater impact frequencies and acceleration magnitudes in games than in practice, similar to findings of high school and college football studies.^4^,^7^,^9^,^29^ For example, a group of high school players, experienced a mean linear acceleration magnitude of 23 g in practices and 25 g in games while the players in this study had a mean linear acceleration magnitude of 22 g in practices and 23 g in games.^5^ With regard to impact frequency, players in this study experienced a similar number of impacts per practice as per game. The rate of impact in practice was similar to the 9.2 impacts per practice that Broglio *et al*.^5^ reported for high school football players. However, the high school players sustained 24.5 impacts per game. These data suggest that high school players experience fewer impacts in practice than in games, while the 9–12 year old players in this study had roughly equal numbers of impacts per session for the two session types.

Substantial differences in impact frequency were observed between team A and the other two teams. For the entire season, players on team A experienced an average of 37–46% fewer impacts than players on teams B and C, though only the difference between teams A and B was statistically significant. This difference is largely due to players on teams B and C participating in 2.1–2.3 times more contact practices than players on team A. The average number of games each player participated in was nearly the same for all three teams, and team A actually had the highest average number of impacts per game at 15.2. Team B and C players averaged 9.9 and 9.6 impacts per game, respectively. Since team A had fewer players than the other two teams, their players may have had more playing time leading to more impacts per game, though other factors such as playing style or skill may have also played a role. For practices, team A players averaged just 6.2 impacts per session compared to 12.9 and 9.5 for teams B and C. Furthermore, players from teams B and C participated in twice as many practice sessions as those from team A. As a result of the higher rate of impact in practices and greater number of practices, team B and C players experienced 219 and 175 impacts during practices, while team A players averaged 48 impacts.

Several factors may have played a role in reducing the head impact exposure observed in team A players relative to teams B and C in this study. First, Pop Warner mandated two rule changes for the 2012 football season that applied to all of their affiliates: (1) a mandatory minimum play rule, where coaches are required to give each player a certain amount of playing time, and (2) a limit on contact in practice, where no more than one-third of weekly practice time and no more than 40 min of a single session can involve contact drills.^28^ While no team in this study was affiliated with Pop Warner, the league in which team A competed enforced the same rule changes, whereas teams B and C had no such restrictions. Second, special teams plays, including kickoffs and punts, were live plays for teams B and C, similar to high school, college, and professional football. Alternatively, team A’s special teams plays were controlled situations where no contact was allowed. Data from previous studies suggest that players on special teams are more susceptible to large magnitude head accelerations, which may lead to higher incidence of concussion on these plays.^2^,^18^,^21^ Third, all three teams played approximately the same number of games during the season, but teams B and C played 11 and 12 week seasons while team A had a 9 week season. With more time between games, teams generally practice at a higher frequency and intensity. Fourth, player skill, athleticism, and maturity could have implications on the level of exposure. Even within teams, variability among players is apparent, with some players experiencing substantially more impacts than the team average. No significant differences were found in game acceleration magnitudes or impact frequency, suggesting practice differences were not due to player differences among teams. Instrumented players ranged from experiencing 72 to 585 head impacts. Fifth, coaching style has major influence on factors such as the types of drills used in practice and the plays called in games. These coaching variations would likely contribute to the differences in the head impact exposure that players experienced.

Two of the impacts (A8 and B4) associated with diagnosed concussions were substantially greater than the player’s season 95th percentile linear acceleration magnitude. Furthermore, the acceleration magnitudes were consistent with concussive values reported in previous studies, albeit at the lower end of the range.^16^,^25^,^27^ For player A8, the impact was the third highest linear acceleration magnitude he experienced during the season and second highest magnitude resulting from an impact to the front of the helmet. The two highest magnitude impacts that this player experienced were similar in magnitude to the concussive impact. For player B4, the concussive impact was his highest magnitude impact to the back of the helmet for the season. This player also accumulated the third highest number of impacts during the season among all study participants. The third impact associated with a concussion (C18) was in the top 20% of linear acceleration magnitudes for that player throughout the season. Although the acceleration magnitude was relatively low for a concussion, it was the player’s second highest magnitude resulting from an impact to the side of the helmet.

The data collected in this study may have applications towards improving the safety of youth football through rule changes, coach training, and equipment design. Prior to the 2012 season, many youth football organizations, including the league in which team A competed, modified rules, and provided coaches with practice guidelines to reduce head impacts in practice. The data collected in this study suggest that head impact exposure over the course of a season can be reduced significantly by limiting contact in practices to levels below those experienced in games. In addition to guiding future rules for youth football, this study can be used to aid designers in developing youth-specific football helmets that may be able to better reduce head accelerations due to head impacts for young football players. Impact location, frequency, and head acceleration magnitudes can be used to optimize helmet padding to maximize protection while keeping factors such as helmet size and mass to age appropriate levels.

A number of limitations should be noted about this study. First, the HIT system used for data collection is associated with some measurement error for linear and rotational acceleration. On average, the HIT system overestimates linear acceleration by 1% and rotational acceleration by 6% when compared to the Hybrid III headform. The correlation between the HIT system and Hybrid III measurements of head acceleration is *R* ^2^ = 0.903 for linear acceleration and *R* ^2^ = 0.528 for rotational acceleration.^1^ Individual data points have uncertainty values due to random error as well; however, the analysis presented here primarily examined distributions of data sets, rather than individual points. Uncertainty values that account for the random error are included with the three concussive data points presented. Second, this study followed three teams consisting of 9–12 year old players with a total of 50 players with large variations in head impact exposure among the different teams and players. Head impact exposure is likely dependent on other factors, in addition to age.

Real-time head impact kinematic data were collected from youth football players, age 9–12 years, during practice and game sessions for an entire season. The data show, on average, that players experienced greater head impact exposure, through more frequent and higher magnitude impacts, than 7–8 year old players, but less than that of high school players. Furthermore, players experienced similar levels of head impact exposure in practice and game sessions on a per-session basis. The vast majority of head impacts recorded in both games and practices were below acceleration magnitudes generally associated with concussions; though, some high magnitude impacts, similar to those seen among older players, did occur. The data presented in this study suggest that head impact exposure at the youth level may effectively be reduced by limiting contact in practices. Future studies are required to determine how rule modifications, coaching style, and other factors influence player impact exposure in practice. Furthermore, additional research is required to determine how reducing head impact exposure in practice affects concussion risk in youth football. Researcher should continue to collect head impact kinematic data in youth football across all age groups to establish the level of head impact exposure a typical player experiences, in a season and career, in order to improve player safety in youth football.
